# Spatial Orientation Assessment in the Elderly: A Comprehensive Review of Current Tests

**DOI:** 10.3390/brainsci14090898

**Published:** 2024-09-05

**Authors:** Panagiota Tragantzopoulou, Vaitsa Giannouli

**Affiliations:** 1 School of Social Sciences, University of Westminster, London W1B 2HW, UK; 2 School of Psychology, Aristotle University of Thessaloniki, 54124 Thessaloniki, Greece; giannouliv@hotmail.com

**Keywords:** spatial orientation, spatial navigation, assessment, aging, neuropsychology

## Abstract

Spatial orientation and navigation are complex cognitive functions that integrate sensory information, attention, and memory, enabling individuals to locate themselves in their environment. These abilities decline with age, signaling cognitive impairment in neurological patients, and significantly limit the autonomy of the elderly. Current neuropsychological assessments fall short in accurately measuring everyday wayfinding abilities, particularly in borderline cases of cognitive decline. This paper reviews various neuropsychological assessments, including Benton’s Judgment of Line Orientation Test, the Almeria Spatial Memory Recognition Test, the Spatial Span subtest from the Wechsler Memory Scale, and the Spatial Orientation in Immersive Virtual Environment Maze Test, evaluating their effectiveness in delineating spatial orientation and navigation skills. The review identifies significant gaps in the validity and reliability of these tests, particularly in their shortened versions, and highlights the potential of virtual reality environments as promising tools for improving diagnostic precision. The findings underscore the need for further research to refine these tools, ensuring they accurately capture cognitive decline and improve the differential diagnosis of neurodegenerative conditions like Alzheimer’s disease. Such advancements hold promise for enhancing the quality of care and autonomy for the elderly.

## 1. Introduction

Cognitive abilities such as executive function, attention, working memory, verbal and visual explicit memory, and processing speed are particularly susceptible to age-related deterioration [1]. In addition to these cognitive skills, spatial orientation, a critical ability for personal independence and daily functioning, has been observed to decline, resulting in diminished performance in various spatial tasks, including cognitive mapping and spatial location [2,3]. Spatial orientation is considered an advanced cognitive function because it requires the integration of multiple cognitive processes. This ability enables individuals to navigate both familiar and unfamiliar environments. More precisely, it involves the processing of visual, proprioceptive, vestibular, and somatosensory information, as well as the capacity to encode and recall spatial information and plan movements [4,5].

Spatial Working Memory (SWM) is a critical cognitive system that temporarily holds and manipulates information about spatial locations and orientations [6]. It plays a vital role in everyday activities such as navigation, problem-solving, and mental arithmetic [7]. Research has linked SWM to genetic predispositions for serious neuropsychiatric disorders like schizophrenia and identified it as one of the first cognitive functions to be affected in various dementias [8,9]. Understanding the cognitive mechanisms and neural bases of SWM has proven challenging. It involves a range of processes, including eye movements, attention, and higher-level cognitive functions, all of which contribute to early spatial information processing [10]. However, in many neuropsychological assessments, SWM is often treated as a single, composite ability, overlooking the complex and intertwined processes that contribute to its function. It should also be mentioned that spatial navigation and SWM, though closely related, are distinct constructs. Spatial navigation encompasses a broad range of skills related to moving through and understanding an environment, while SWM is specifically concerned with the short-term storage and manipulation of spatial information [4]. Therefore, SWM supports spatial navigation by providing the necessary short-term storage and manipulation of spatial information needed to make decisions and plan movements [7]. Further, they rely on different neural mechanisms. The hippocampus plays a crucial role in spatial navigation by supporting the encoding and retrieval of spatial memory [4,5]. In contrast, SWM is predominantly associated with the prefrontal cortex, which is critical for the short-term storage and manipulation of spatial information [6].

A crucial aspect of cognitive decline in aging is topographical disorientation (TD), which involves difficulties in acquiring spatial information in new environments and navigating familiar ones, such as one’s neighborhood or home [11]. Recent research has found that TD is a significant risk factor for developing mild cognitive impairment (MCI) and its amnestic subtype (aMCI) [11]. However, this significant association was observed only when the researchers used objective measures to assess TD. Navigation encompasses two types of spatial orientation: egocentric and allocentric. Egocentric orientation, which is mediated by the parietal lobes and subcortical regions, involves orienting oneself based on one’s own position in the environment [12]. In contrast, allocentric orientation, governed by the hippocampus, relies on environmental landmarks as reference points to navigate toward target destinations and plan actions [12]. These two reference systems function collaboratively to facilitate navigation. In daily life, individuals typically employ both frameworks, dynamically switching between and integrating various spatial strategies according to the specific demands of their surroundings. Studies collectively suggest that aging impacts spatial cognition in a selective and nuanced manner, with different spatial components and strategies showing varying degrees of decline. In a study involving 140 participants, evenly divided by sex and categorized into seven age groups ranging from 20 to 89 years, findings indicate that aging selectively affects spatial processing [13]. Specifically, significant declines in egocentric components were observed beginning in the 70s, while allocentric components appeared to remain relatively intact [13]. Conversely, a systematic review revealed that while egocentric strategies in spatial navigation are generally preserved, there are notable impairments in allocentric strategies and the ability to switch between spatial frames of reference [14]. These results imply that egocentric and allocentric spatial processes are supported by distinct neural regions, each exhibiting different degrees of susceptibility to the effects of aging. From a clinical perspective, these findings underscore the necessity of differentiating between spatial components that decline with normal aging and those that remain stable. 

Early and comprehensive evaluation of spatial disorientation is crucial, as timely detection of these impairments can improve the likelihood of recognizing the beginning stages of pathological cognitive decline. Tests for spatial orientation have shown significant sensitivity in identifying both the onset and progression of cognitive impairments, establishing them as valuable indicators [15]. However, there is currently no definitive standard test for evaluating spatial orientation, and existing traditional paper-and-pencil assessments lack the ecological validity and sensitivity required to accurately detect spatial disorientation. Virtual Reality (VR), a computer-generated technology, has demonstrated its effectiveness in assessing cognitive functions and is increasingly being utilized in assessments [16].

The primary aim of this paper is to review various tests currently employed to assess spatial orientation in the elderly, focusing on the methodologies involved, their strengths, and their limitations. By providing a comprehensive examination of these assessments, the paper seeks to highlight how these tools measure spatial orientation. The review will cover both traditional paper-and-pencil tests and modern VR applications, comparing their practical applicability. The selection of tests included in this review was based on a thorough search of scientific literature in key databases, including PubMed and Google Scholar, using relevant keywords such as “spatial orientation”, “spatial navigation”, and “cognitive aging.” While no formal guidelines like PRISMA were employed, the review emphasizes tests that are widely recognized in research and clinical practice. Moreover, the paper incorporates relevant research findings to contextualize the effectiveness of these assessments and discuss the extent to which they can reliably detect the onset and progression of cognitive impairments. This critical analysis aims to offer insights into the current landscape of spatial orientation assessment, identifying areas for improvement and potential directions for future research to enhance early detection and intervention strategies for cognitive decline in the elderly.

## 2. Assessment Tools

### 2.1. Benton’s Judgment of Line Orientation

The Benton Judgment of Line Orientation Test (BJLO) is a widely recognized neuropsychological assessment tool, frequently employed in both clinical and research contexts to evaluate spatial perception and orientation. The BJLO test comprises line segments of various orientations that must be matched to a set of longer lines on a response card [17]. Each test page displays two partial line segments, and the participant must match their orientations to those on a multiple-choice response card, which contains 11 full lines spaced 18 degrees apart and arranged in a semicircle. The partial line segments represent the proximal (“low”, “L”), middle (“M”), or distal (“high”, “H”) third of the full lines. Participants are initially given five sample items, with corrections provided for any errors, followed by 30 test items presented without feedback [17]. Scoring is based on the number of correct responses, with adjustments for age and gender factored into the total score. Scores categorize individuals as normal, borderline (mild), moderate, or severely impaired [17]. The BJLO test is advantageous due to its purely visual nature, requiring minimal motor response, and avoiding confounding factors such as constructional praxis or information processing speed, which can affect other visuospatial tests [18]. Further, the test exhibits robust psychometric properties, including high test-retest reliability [19], and demonstrates strong neuropsychological construct validity, as evidenced by neuroanatomical localization studies [20]. However, the BJLO test has notable disadvantages. The full set of 30 items can be particularly challenging for patients with moderate to severe brain damage and may induce fatigue in psychiatric patients and healthy controls, especially when included in a larger battery of tests and considering that the test evaluates a fundamental visuospatial skill [21,22]. Since the items are arranged in order of increasing difficulty, participants encounter the most challenging items when they are likely to be most fatigued.

In response to these limitations, several researchers have attempted to examine the clinical accuracy of shorter versions of the test. For instance, Woodard et al. [23] divided the original full version into two parts by assigning odd and even items to separate groups, creating shorter forms based on the difficulty progression of the original 30 items. However, Woodard et al. [23] found that the frequency distribution of errors revealed prediction errors as high as six points. Considering that the maximum score on the JLO test is 30, this represents a significant margin of error, encompassing 20% of the total test items. Similarly, Qualls et al. [24] analyzed the difficulty levels of each item to create two parallel versions, Q and S, and used a Latin Square randomization method to assign items to each version. Both new sets, O/E and Q/S, maintained a roughly ascending order of item difficulty. Nonetheless, the authors found that their short forms misclassified 10% of the study participants and thus suggested using them primarily as screening tools for visuospatial impairment [24].

Calamia and colleagues [25] tested a more flexible version. In their version, the examiner starts at the sixteenth most challenging item and determines a basal level (six items passed) and a ceiling (six items failed). This approach reduces the number of items needed to achieve scores comparable to the full form by almost one-third. Once a basal level is set and a patient surpasses the cut-off score for intact performance, the test can be concluded. For example, if the cut-off score is 21 and a patient correctly answers the first six items on the short form, their predicted score will be at least 21, allowing for the termination of the test. Conversely, if a patient misses the initial item and continues to miss subsequent items in reverse order, reaching a point where a score above 21 is unachievable, the test can also be concluded early. This flexible short version was found to optimize the balance between reducing test length and maintaining measurement accuracy [25]. However, a limitation of this study is that the shortened version was not tested on a new set of participants, such as individuals who had never taken the BJLO test before. Therefore, the results of this study could have been influenced by the sample that was used and the design of the study.

### 2.2. Spatial Span Subtest from the Wechsler Memory Scale

Visuospatial processing involves both the manipulation and short-term retention of visual information. It includes visual working memory and the consolidation process, which transforms fleeting perceptual representations into durable working memory [26]. Aging and dementing disorders, such as Alzheimer’s disease (AD) and vascular dementia (VaD), significantly affect the ability to process and store visuospatial information [27]. To assess this impairment, the Spatial Span subtest from the Wechsler Memory Scale is a commonly used measure. This subtest evaluates an individual’s capacity to remember and replicate a sequence of spatial locations held in visual working memory [28]. As part of a comprehensive neuropsychological assessment, the Spatial Span (Forwards and Backward) test uses a three-dimensional board with 10 blocks to form various spatial patterns. For the forward test, the examiner points to the blocks in a specific order, and the participant must replicate the sequence from memory. For the backward test, the examiner points to the blocks in a set pattern, and the participant must recreate the sequence in reverse order. Each test starts with sequences of just two blocks, and participants are given two tries for each sequence length. The sequences can be as long as eight blocks. The test ends if the participant fails both attempts for a sequence length. Points are awarded for each correctly completed sequence, with a total of 32 possible points for the Spatial Span subtest: 16 points for the Spatial Span Forward and 16 points for the Spatial Span Backward [29].

A study investigated the impact of cognitive impairment and its severity on performance in the Spatial Span task, which was part of a comprehensive neuropsychological battery administered to 538 elderly individuals aged 65–89. The participants were categorized into groups based on their cognitive status: AD, VaD, aMCI, Non-Amnestic Mild Cognitive Impairment (naMCI), and cognitively normal. The results indicated that performance on the Spatial Span task declines as cognitive impairment becomes more severe. Notably, age, which has been previously shown to affect visuospatial processing, did not significantly influence Spatial Span performance in this study. Both age and gender were weakly related to Spatial Span performance, and Spatial Span is more sensitive to cognitive impairment, such as dementia, than to normal aging. Additionally, the study found that different aspects of the Spatial Span task have varying sensitivities to cognitive impairment. Performance on the Spatial Span Forward task remained relatively stable regardless of the level of impairment. In contrast, the Spatial Span Backward task showed greater sensitivity to severity, with marked declines especially notable when compared to cognitively normal individuals [29]. This suggests that the Spatial Span Backward task is more challenging due to its novelty and the higher demand for complex working memory skills, such as manipulation, compared to the more familiar Spatial Span Forward task. Nonetheless, a secondary analysis of 1030 participants between the ages of 16 and 89 revealed that the rate of age-related decline was equivalent for both forward and backward span tasks [30]. This finding suggests that both types of span tasks rely on central executive resources to a similar extent for successful performance and that age might affect both tasks equivalently.

### 2.3. Corsi Block-Tapping Test

The Corsi block-tapping test (CBT) [31] comprises nine blocks arranged randomly on a flat surface. The examiner taps a specific sequence of blocks while the participant observes. In the forward version of the test, the participant replicates the sequence as demonstrated, whereas in the backward version, the participant repeats the sequence in reverse order. The number of blocks in the sequence begins small and increases with each correctly replicated sequence until the participant fails to reproduce a sequence. The longest successfully repeated sequence, known as the Corsi span, is recorded. For adults, the average forward Corsi span ranges between five and seven blocks [32]. Digital versions of the test have been developed where the physical blocks are replaced with digital squares and the tapping action has been changed to a color change in the blocks while participants use a computer mouse to select the blocks. Additionally, the CBT was scaled up to incorporate walking versions, which allowed researchers to examine different aspects of spatial memory involved in wayfinding [33].

The CBT is widely used in neuropsychological and medical research to study conditions like acquired brain injury, dementia, Parkinson’s disease, and AD [34]. A previous study used the CBT to investigate spatial working memory in patients with AD. After evaluating 30 elderly control subjects and 30 patients with probable AD, the study found significant differences in spatial memory performance between AD patients with moderate dementia and the control group, while those with mild dementia did not show significant differences compared to controls [35]. This suggests that spatial memory may remain relatively unaffected in the early stages of AD but declines as the disease progresses to moderate dementia. The absence of significant differences in mild dementia could be due to the small sample size or the test itself. The CBT has faced several methodological criticisms. A major issue is the lack of standardization across studies. Researchers often modify key aspects of the test without providing justifications, leading to inconsistencies that make it difficult to compare results from different studies [34]. This inconsistency is compounded by the fact that many studies do not report important details about how they administered the CBT, which further complicates the interpretation of results [34].

Digital versions of the CBT offer significant improvements over the traditional physical versions, addressing some of the methodological issues. One of the main advantages is increased reliability. Digital CBTs can be programmed to have consistent settings, ensuring that test conditions are the same for all participants. This consistency enhances the reliability of test results when repeated over time [36]. Additionally, digital CBTs can have fixed parameters, which reduces variability in test administration and facilitates the standardization of the test [36]. This standardization makes it easier to compare results across different studies, thereby improving the overall robustness of research findings. However, digital CBTs are not without their challenges. The variability in how the test is displayed on different devices, such as tablets, computers, and virtual reality headsets, can influence how participants perform, introducing a new source of variability [34]. Despite these challenges, digital CBTs still represent a significant step forward in addressing the methodological limitations of the traditional CBT, offering a more reliable and standardized approach to assessing spatial memory.

### 2.4. Money Road-Map Test

Right-left confusion in adults is an intriguing aspect of spatial cognition and clinical neuropsychology. The ability to distinguish between right and left is essential for many everyday activities and typically develops through childhood, becoming adult-like around the age of 12 [37]. This skill relies on several higher cognitive functions, including the integration of sensory information, language, memory, and visuospatial processing [38]. Interestingly, many healthy adults still experience difficulties with right-left discrimination in various contexts. Self-evaluation studies have shown that women often report more susceptibility to right-left confusion than men [39,40]. One notable tool used to measure right-left orientation in the elderly is the Money Road-Map Test (MRMT). This test involves a two-dimensional city map on a single sheet of paper. The map features a winding path with 32 right and left turns at various angles [41]. The experimenter traces a winding path through city streets, and participants must quickly indicate whether a right or left turn is needed at each corner. Participants are not allowed to rotate the map, which means they must make some right-left judgments from a reversed perspective. The test records both the accuracy of the right-left responses and the time taken to complete the route.

Morganti et al. [42] adapted the MRMT into a computer-based task where participants can view the environment on a computer screen and navigate using a joystick or similar device. In this study, researchers investigated spatial orientation difficulties in AD patients, focusing on their ability to convert spatial knowledge from an allocentric (map-based) perspective to an egocentric (self-centered) perspective. One of the tests they employed was the VR-RMT, with 26 AD patients and 26 healthy elderly participants. Results revealed that AD patients were significantly impaired in this task compared to healthy participants [42]. While healthy individuals effectively maintained and updated their spatial perspective using the allocentric map, AD patients had substantial difficulty converting this information into egocentric actions. This impairment was particularly pronounced when participants had to continuously adjust their position based on the turns they made in the virtual environment. The findings suggest that AD patients struggle with tasks that require the translation of allocentric spatial information into egocentric perspectives, likely due to early degeneration of brain regions such as the hippocampus and retrosplenial cortex. This specific impairment in spatial orientation highlights the potential of VR-based tasks in diagnosing and understanding spatial disorientation in AD. It was also considered that the VR-RMT, by providing real-time external visual feedback, offers an added advantage in assessing spatial orientation abilities compared to traditional paper-based tests [42]. However, it also presents additional challenges for AD patients, as errors in turns can lead to further disorientation, affecting subsequent navigation decisions.

Comparing the traditional and the virtual reality-based test among 83 healthy right-handed volunteers aged from 30 to 80 years, scholars found that the VR-RMT was significantly more complex and challenging for participants compared to the MRMT [43]. This complexity was evident in the dual-task nature of VR-RMT, which required participants to alternate between making decisions on a paper map and executing those decisions in a virtual environment. This continuous shift in focus and perspective switch made the VR-RMT more mentally demanding. A notable age-related decline in performance was observed, with older participants experiencing more difficulty in the VR-RMT [43]. In the MRMT, participants relied on imagining spatial transformations, whereas in the VR-RMT, they physically enacted these transformations, leading to different cognitive demands. The VR-RMT required continuous sensorimotor coupling, which appeared to be more effortful than the imaginative process in the MRMT. Additionally, errors in the VR-RMT had a compounding effect, significantly impacting subsequent navigation decisions and overall task performance [43]. These findings highlight the greater difficulty of VR-based assessments, particularly for older adults, and underscore the importance of considering age and technological familiarity when designing and interpreting virtual reality-based cognitive tests.

### 2.5. The Spatial Orientation in Immersive Virtual Environment Maze Test

The Spatial Orientation Test in an Immersive Virtual Environment (SOIVET) system [44] is an advanced method designed to assess spatial navigation skills using virtual reality. It includes two tasks: the SOIVET-Maze task and the SOIVET-Route task, both of which leverage immersive VR technology to create a realistic and engaging experience. The SOIVET-Maze task, based on the traditional MRMT, evaluates participants’ ability to switch between an overhead map view (allocentric) and a first-person view (egocentric) as they navigate a virtual maze. Participants wear a headset and use hand-held controls, guided by a high-performance computer. Before the actual test, they undergo a training stage to familiarize themselves with the VR system. In the task, participants navigate through a maze with identical walls, following a path shown on a map at the bottom of the screen, making left or right turns as needed. The system records the number of correct turns, using the same scoring method as the paper-based MRMT. The VR setup stimulates visual, vestibular, and somatosensory senses, engaging brain areas related to real-life navigation, while the evaluator monitors the participant’s progress in real-time. The SOIVET system proved to be an effective tool for assessing spatial orientation in older adults, both with and without mild cognitive impairment (MCI) [44,45]. This innovative approach provides a more dynamic and immersive way to evaluate spatial orientation, offering insights that traditional paper-based tests might miss.

### 2.6. Almeria Spatial Memory Recognition Test

The Almeria Spatial Memory Recognition Test (ASMRT) [46] evaluates spatial memory through a virtual room containing nine brown boxes arranged in three rows. In the sample phase, participants are shown an image where one box is green, and they have ten seconds to memorize its location. In the recognition phase, ten images are presented one by one, each containing a green box among brown boxes, and participants must decide if the green box is in the same position as in the sample image. Evaluating and deciding on images from various perspectives may necessitate converting between egocentric and allocentric spatial representations. Half of these images are correct, and responses are recorded without time pressure, typically taking less than a minute per trial. The test includes three difficulty levels, requiring participants to remember one, two, or three green boxes in the sample phase, although each recognition image contains only one green box. This setup provides a comprehensive assessment of spatial memory by evaluating participants’ ability to memorize and recognize box positions under varying conditions and levels of difficulty. The test does not require elaborate procedures or specialized equipment, making it convenient for use in medical consultations. Additionally, this ease of application makes the ASMRT well-suited for brain imaging studies, as it can be integrated into these studies without adding complexity [46].

The ASMRT has been used in studies to compare younger and older adults. Castillo-Escamilla and colleagues [47] utilized the ASMRT to investigate how age, sex, and task difficulty influence spatial recognition performance. The study highlighted that spatial recognition is affected by task difficulty, with increased errors for older adults when more boxes were to be remembered and anticlockwise rotation was used. Additionally, sex differences emerged in the most challenging conditions, with men generally outperforming women. However, the study had limitations, including smaller sample sizes, when analyzing combined age and sex factors, and the inability to measure precise response times due to manual registration of responses.

A later study, however, that used the ASMRT to investigate how aging affects spatial recognition skills and whether the test could reveal differences related to age and sex, found no significant sex differences in performance [48]. This lack of sex difference in the elderly may be due to the similar challenges both sexes face with allocentric strategies in old age. Nonetheless, the findings of the study indicated that older adults, specifically those aged 70–79, perform worse on the ASMRT compared to younger participants aged 50–59, showing more errors as task difficulty increased [48]. This decrease in performance among older adults suggests that declines in executive functions and attentional resources contribute to poorer spatial recognition.

### 2.7. Τhe Virtual Supermarket Test

In recent years, computerized cognitive screening tests have garnered significant attention from researchers. One such tool that has attracted considerable interest is the Virtual Supermarket Test (VST). Developed by the Center for Research & Technology Hellas/Information Technologies Institute (CERTH/ITI) in collaboration with the Greek Association of Alzheimer’s Disease and Related Disorders (GAADRD), the VST is designed to evaluate cognitive abilities related to visual and verbal memory, spatial navigation, attention, executive function, and MCI [49,50].

The VST assesses spatial navigation capabilities, including egocentric orientation, allocentric orientation, and heading direction [49]. In the form of a game, the VST simulates a daily shopping task. During the exercise, a shopping list appears in the upper right corner of the screen. Participants must locate the listed items, place them in a shopping cart, bring them to the cashier, and pay the correct amount. Navigation within the virtual supermarket is achieved by touching green footprints on the screen to move the shopping cart. To prevent learning effects from repeated trials, the items on the shopping list are randomly generated each time. This active exploration of the virtual environment enhances learning and memory [49].

The VST can be administered on both computers and tablet devices and is available in both self-administered and examiner-administered versions. It has also been translated and adapted for use in Turkish. This tool has been validated and proven effective in detecting MCI in older adults in studies involving Turkish and Greek participants, regardless of whether they report subjective memory complaints [49,50,51]. Furthermore, a study involving 24 healthy older adults with subjective cognitive decline and 33 patients diagnosed with MCI indicated good usability, which was not influenced by variables such as age, education, familiarity with touch devices, or MCI diagnosis [52]. Lastly, in a preclinical AD population, the VST demonstrated good test-retest reliability [53], although future research should further assess its reliability in larger samples.

### 2.8. Sea Hero Quest

An additional novel spatial navigation measure is the Sea Hero Quest (SHQ) which is a mobile and tablet game designed to assess spatial navigation abilities in both laboratory and large-scale online settings. In this game, players navigate a boat through a virtual ocean environment to locate and photograph sea creatures [54]. SHQ consists of two types of levels: wayfinding and path integration. In the wayfinding levels, players are initially shown a map displaying the starting point and the locations of numbered checkpoints. They study the map for as long as needed, then tap the screen to make the map disappear. Players must then navigate the boat to the checkpoints in order, relying on their memory of the map [55]. This task involves complex cognitive processes, including map interpretation, route planning, route memory, progress monitoring, route updating, and transforming a bird’s-eye view into an egocentric perspective for navigation [54]. In the path-integration levels, participants are not given a map but are asked to navigate a winding river to find a flare gun. Upon finding the flare gun, the boat rotates 180° clockwise, and participants must shoot the gun in the direction they believe the starting point is located, choosing from three options (right, center, left). This level measures egocentric orientation by requiring participants to encode and recall the starting location relative to their current position.

Research using SHQ in older adults is limited, but there are studies that have assessed its utility and validity in measuring spatial ability. For instance, Spiers et al. [56] found that spatial ability generally declines with age. However, surprisingly, after the age of 70, performance in spatial ability tests appeared to improve. This unexpected result is likely due to the fact that older adults participating in such studies, particularly those playing video games for research on their smartphones or tablets, are probably among the most cognitively capable in their age group. This finding highlights the importance of considering selection bias when measuring cognitive abilities in older adults and generalizing the results. The same study also found that males generally have a slight advantage over females in spatial ability. While scholars expressed concerns that SHQ might not fully reflect real-world navigation skills, a comparison with a similar navigation task in the streets of London and Paris suggested that SHQ is indeed a good tool for measuring real-world navigation skills [56]. This comparison indicates that SHQ can effectively capture aspects of spatial navigation performance relevant to real-world contexts. Regarding test-retest reliability, it was found that some aspects of the SHQ game, such as the distance traveled by participants, show moderate test-retest reliability [56]. This means that participants’ performance on this measure can be relatively consistent over an 18-month period. However, other measures, such as the duration of the SHQ game, show only low test-retest reliability, indicating more variability in participants’ performance over time [56]. The real-world ecological validity of the test has also been indicated by a study on 60 young adults, proposing SHQ to be a significant advancement toward creating digital cognitive assessments that are controllable, sensitive, safe, low-cost, and easy to administer [57]. It is important to note that this study did not include any adults within the older age group. Further research is needed to test the reliability and validity of SHQ in older populations.

### 2.9. Detour Navigation Test

The Detour Navigation Test (DNT) is an innovative assessment designed to evaluate spatial navigation abilities in participants through real-world and virtual environments. These environments can include video games, computer simulations, physical mazes, or naturalistic community settings. The primary objective of the DNT is for participants to navigate from a designated starting point to a specified destination while encountering obstacles or detours that necessitate adjustments to their planned route [58]. Participants are initially provided with clear instructions regarding the starting point, the target destination, and the navigation task. As they begin the test, they move through the environment and encounter various obstacles or barriers. These obstacles compel them to find alternative paths, demonstrating their capacity to adapt to changing conditions. The test concludes when the participant successfully reaches the target destination. Participants’ performance is assessed based on various metrics such as the time taken to complete the task, the distance traveled, the number of errors made, and overall navigation efficiency.

Puthusseryppady and colleagues [58] designed a study aiming to examine the spatial navigation abilities of AD patients and determine if VR navigation performance could predict which patients are at high risk for spatial disorientation in the community. To accomplish these objectives, three VR tests were utilized: the VST, SHQ, and DNT. The results revealed that AD patients between 50 and 80 years of age showed significant impairments in spatial navigation when compared to age- and gender-matched controls, in both VR environments and real-world settings. Specifically, the patients displayed deficits in performance on the VST, SHQ, and DNT. However, these VR measures did not reliably predict which patients were at the highest risk of spatial disorientation in real-world situations [58]. This study underscores the importance of future research aimed at developing VR-based tests that can accurately identify AD patients who are at a high risk of experiencing spatial disorientation in real-world contexts. While some additional studies have implemented a modified version of the DNT in various populations [59,60], the test’s use by the scientific community appears to be limited, and definitive conclusions about its reliability and usability in the elderly cannot be drawn.

### 2.10. Morris Water Maze

The Morris water maze (MWM) is a test developed by the British neuroscientist Morris in 1981 to study spatial learning and memory. It is popular among psychologists and neuroscientists for use with both animals, such as rodents [61], and humans in virtual simulations [62]. The widespread use of the MWM can be attributed to several factors. More specifically, it eliminates the need for pre-training, demonstrates consistent reliability across various tank configurations, and serves as an effective measure of hippocampus-dependent spatial navigation and memory [63,64]. It is also specifically designed to test place learning and is not affected much by different motivations due to genetic, pharmacological, nutritional, toxicological, or brain damage treatments [64].

Morris outlined the basic procedures in 1984 and added more detailed methods for related learning and memory assessments later. The MWM is not a typical maze with walls and paths. Instead, it is a circular pool filled halfway with water and kept as featureless as possible [61]. The animal, as well as the human in the virtual environment, must find a hidden platform submerged just below the water surface, always in the same place. The platform is hidden by making the water opaque or matching the platform’s color to the water, so that the participant cannot see it easily.

The test has been used in studies trying to compare younger and older adults’ spatial navigation skills. More specifically, in 2021, researchers explored how older adults navigate and remember spatial information compared to younger adults using a virtual analog of the MWM [65]. The study found that older adults were generally less accurate in remembering target locations than younger adults. However, both age groups performed similarly when tested from the same or different viewpoints, indicating that older adults could still generalize their spatial memory when seeing things from a new perspective. Additionally, both groups used a mix of strategies based on multiple landmarks and single landmarks to navigate, showing that older adults can still effectively use these strategies despite having less precise spatial memories [65]. Although these results suggest that age-related changes might affect how people navigate and the test was able to identify some differences, conclusions cannot be made as the sample size (12 younger adults and 15 older adults) was small. An additional study that tested navigation performance among 213 healthy adults, aged 18 to 77, twice over two years, used the MWM [66]. Initially, participants performed similarly in both testing sessions, but their improvement during each session declined over time. Older adults had more difficulty navigating, taking longer paths, which was linked to higher blood pressure, smaller brain regions (cerebellum and caudate), and higher iron content in the caudate. The complexity of their navigation paths became improved with practice, but older adults showed less improvement [66]. In reviewing this study, several significant limitations must be highlighted. First, while the researchers reported differential patterns of longitudinal changes in navigation indices and their neural correlates, they did not test competing hypotheses within a single comprehensive model. This limitation arises partly from the modest sample size, which was further reduced by non-random attrition—39% of the original sample did not return for follow-up assessments. Participants may have found the test too challenging or frustrating, leading them to opt out of subsequent assessments. The cognitive load required for difficult tasks can be particularly burdensome for elderly participants.

### 2.11. 4 Mountains Test

The 4 Mountains Test (4MT) [67] is an efficient assessment of working allocentric spatial memory, evaluating the ability to recall spatial configurations from altered viewpoints, which reflects the role of the hippocampus in spatial cognition. Each test item consists of five images of computer-generated landscapes. Participants first view a sample image and then must select the corresponding target image from four alternatives, each depicting the same landscape from a different angle. The three incorrect options (foils) feature landscapes with systematically different topographies. Each landscape includes similar topographical elements: a ground plane with small undulations, a semi-circular mountain range defining the horizon, and four prominent mountains of varying shapes and sizes. These landscapes are designed to have unique global topographies while sharing local features with the foil images, making it difficult to rely on nonspatial memory strategies. The sample and target images are generated from the same landscape but are viewed from different camera angles, promoting the use of allocentric spatial strategies over egocentric or visual strategies. Additionally, nonspatial features such as lighting, colors, textures, and weather conditions are varied between the sample and test images to further discourage visual pattern matching. Each test item comprises a sample image with unique nonspatial features, a target image showing the same landscape from a different angle, and three foil images with distinct topographies but sharing nonspatial features with the target.

Recent studies have demonstrated the 4MT’s effectiveness in distinguishing between MCI patients with and without biomarker evidence of underlying AD, highlighting its potential as a clinical tool for early detection of pre-dementia AD. This utility was further confirmed through its successful application in a cohort of MCI patients recruited from Italian memory clinics, indicating the test’s applicability across different clinical and cultural contexts due to its language-independent design [68]. An additional study that tested the 4MT on people with MCI, some of whom had biomarkers indicating a higher risk of developing AD, as well as on people with mild AD and healthy individuals, showed that the test can distinguish between MCI patients with and without AD biomarkers [69]. It was further concluded that its short duration, ease of administration and scoring, and favorable psychometric properties make it a valuable diagnostic tool for pre-dementia AD [69]. However, the need for precise instruction and practice indicates that further standardization and methodological rigor are necessary to maximize its diagnostic accuracy.

### 2.12. Apple Game

To assess path integration (PI) performance, a novel task called the “Apple Game” [70] has been developed specifically for older adults (≥ 65 years). Participants are required to navigate through a series of trials, each involving three phases. In the start phase, participants locate a basket and memorize its position as the goal location. During the outgoing phase, they navigate toward a variable number of trees, ranging from one to five, with each tree’s distance varying to increase PI difficulty. Upon reaching a tree with an apple, the trees disappear from view. In the incoming phase, participants must find the shortest route back to the original basket location. The included subtasks in the Apple Game evaluate PI under different spatial cue conditions: Pure PI (PPI) relies solely on visual flow without additional cues, Boundary-supported PI (BPI) provides a circular boundary as a cue, and Landmark-supported PI (LPI) includes an intra-maze landmark such as a lighthouse [70]. This setup allows for a comprehensive assessment of spatial navigation abilities under varying conditions.

The Apple Game has been used in a study that investigated how spatial navigation abilities decline in individuals with a genetic risk for AD, even before clinical symptoms appear. Researchers included 145 participants with an average age of 64, who were cognitively normal [71]. They were divided into two groups based on their APOE gene: E4 carriers (who have at least one ε4 allele and are at higher risk for AD) and E3 carriers (who do not have the ε4 allele). The results showed that as participants aged, their navigation errors increased. Specifically, in the simplest version of the task (with no interim locations and no landmarks), E4 carriers older than 70 had significantly higher errors compared to E3 carriers, indicating a decline in their path integration abilities [71]. This decline was mainly due to increased errors in estimating direction rather than distance. When the task included interim locations, all participants over the age of 50 performed poorly, indicating the task’s difficulty level was too high for this age group. However, when landmarks were present, errors were not influenced by the APOE genotype, suggesting that landmark-based navigation declines with age but is not specifically related to AD risk. In summary, the study found that elderly individuals with a genetic risk for AD showed deficits in spatial navigation, particularly in tasks relying on pure path integration without visual landmarks [71]. This deficit likely reflects early AD-related brain changes in the entorhinal cortex, whereas age-related declines in landmark-based navigation were not linked to AD risk.

Based on the findings, we can infer that the Apple Game test is a promising tool for detecting early deficits in spatial navigation, particularly in individuals at higher genetic risk for AD. Despite its potential, the Apple Game test has been scarcely used, even though it was specifically designed to assess spatial navigation abilities in older adults. This underutilization may be due to several factors. Firstly, the task’s complexity, especially in trials with multiple interim locations, may make it challenging for broader application in older populations without extensive training. Additionally, while the Apple Game can effectively highlight specific navigation impairments related to AD, it may not yet be widely recognized or adopted in clinical and research settings focused on aging and cognitive decline. In summary, the Apple Game offers a unique and effective means of assessing spatial navigation in older adults, particularly for identifying early AD-related impairments. However, its limited use to date highlights the need for more extensive validation and wider adoption in both research and clinical practice.

The table below provides a concise summary of the main findings (see Table 1):

## 3. Discussion

Assessing spatial navigation is crucial for understanding cognitive aging and neurodegenerative conditions, particularly AD and other dementias that prominently affect spatial orientation. In AD, spatial disorientation often appears early and can significantly impair daily functioning, making it a key diagnostic marker [27]. Proper assessment of spatial navigation abilities can thus play a vital role in distinguishing AD from other neurodegenerative disorders, such as frontotemporal dementia (FTD) and Lewy body dementia (LBD), where spatial orientation may be affected differently. By improving the accuracy and specificity of spatial navigation assessments, clinicians can better differentiate between these conditions, leading to more tailored interventions and management strategies.

Various tests designed to measure spatial navigation abilities offer diverse approaches and insights, each with distinct strengths and limitations impacting their clinical and research applications. As shown in Table 2, several gaps exist in the current landscape of spatial navigation tests, and addressing these gaps is essential for advancing the field. For example, the BJLO test has shown high error rates when shortened, indicating the need for rigorous validation on larger, more diverse samples to ensure these shorter versions maintain clinical accuracy [23]. Similarly, digital formats of tests like the CBT suffer from a lack of standardization, which complicates the comparability of results across studies. To address this, future research should prioritize standardizing digital versions to ensure consistency in device usage and reporting methods. This involves rigorous testing on larger and more diverse samples of elderly participants, which will provide more robust data on the efficacy and reliability of these tools. Additionally, exploring the application of these tests in various real-world contexts and across different cognitive states will enhance their clinical relevance for older adults and improve the differential diagnosis of neurodegenerative disorders.

The development of traditional tests into digital formats and the use of VR environments represent significant advancements in this field. As this paper has shown, VR environments are promising in detecting declines in spatial orientation in the elderly and discerning between individuals with a genetic risk for AD and MCI patients [34,69,71]. Their applicability across different clinical and cultural contexts due to their language-independent design has also been confirmed [68]. However, the limited research on these VR tools, as highlighted in the evaluation of tests like the SOIVET, underscores the need for more accessible and standardized VR solutions. Developing more affordable versions and conducting validation studies with diverse elderly populations will be crucial steps in integrating VR tools more effectively into clinical practice. Moreover, improving the standardization of digital protocols will enhance the reliability of spatial memory assessments for the elderly by minimizing variability across devices and environments. Greater consistency and transparency in test administration and reporting, particularly for digital and modified versions of traditional tasks, will improve the comparability and robustness of findings. For tools like SHQ and DNT, it is essential to improve test-retest reliability and validity across different populations by refining methodologies and ensuring consistency in performance metrics. Additionally, increasing the standardization and simplifying the administration of tests like the 4MT and the Apple Game will enhance their practical applicability in clinical settings. This includes refining instructions and ensuring that these tests are accessible to a broader range of elderly populations, considering sensory and motor limitations common in this age group.

It is important to note that while some VR tests for spatial navigation have shown promising initial results, their use in research remains limited, hindering the potential for further development and the advancement of methods for detecting declines in the elderly. This scarcity in research can be attributed to factors such as high development costs, the need for specialized equipment, and the steep learning curve associated with VR technology. Additionally, the lack of standardized protocols and scoring systems creates inconsistencies in data collection and interpretation. To reverse this trend, it is crucial to invest in developing more cost-effective VR solutions and establish standardized methodologies. Encouraging collaborations between technology developers and clinical researchers can also help integrate VR tools into clinical practice, ultimately enhancing early detection and intervention strategies for cognitive decline in the elderly. Proper assessment of spatial orientation and navigation in neurodegenerative disorders could significantly improve the differential diagnosis process, enabling earlier and more accurate identification of conditions like AD. Aside from detection, VR environments can also be used to tackle cognitive impairment and train the elderly, providing an engaging platform to enhance various aspects of cognitive functioning [72,73]. Finally, combining various measures, such as the immersive nature of SOIVET and the practical tasks of VST and DNT, could offer a more holistic assessment of spatial navigation abilities in the elderly. Integrating these tools may enhance diagnostic precision and provide a comprehensive evaluation of cognitive function. Such multi-faceted approaches can capture a broader range of spatial abilities, making assessments more robust and reflective of real-world navigation skills.

This review is subject to several limitations that may impact the comprehensiveness and accuracy of its findings. Firstly, the reliance on keyword searches and datasets for identifying relevant tests may have led to the omission of significant studies or innovative tests not captured by these search terms. Additionally, the restriction to English-language publications might have excluded valuable research published in other languages, potentially resulting in an incomplete overview of spatial navigation and orientation tests. The review also did not include a meta-analysis, which means it is primarily qualitative and may not fully represent the overall performance and comparative efficacy of the tests reviewed. Furthermore, due to technological constraints and publication lag, the review may not have encompassed the most recent developments in VR and other digital assessments. Consequently, newer advancements in these areas might not be fully represented, potentially affecting the review’s relevance to current and emerging technologies. Addressing these limitations in future research could provide a more comprehensive understanding of spatial orientation assessments and their applications in clinical settings.

## 4. Conclusions

In conclusion, the current array of spatial navigation tests offers valuable insights into cognitive aging and neurodegenerative conditions in the elderly, but specific advancements are necessary for their effective application in clinical settings. Among the tools reviewed, VR environments show considerable promise due to their ability to provide immersive and ecologically valid assessments of spatial navigation abilities. These VR-based tools have demonstrated strong potential for detecting declines in spatial orientation and differentiating between cognitive states such as MCI and AD, making them particularly useful in clinical practice. The use of VR can also offer a more engaging and interactive approach to assessment, which may improve patient compliance and the accuracy of results. Additionally, traditional tests, despite their current limitations, could offer valuable diagnostic insights when validated and refined.

To effectively translate these findings into clinical practice, a focused effort on standardizing digital protocols and improving test-retest reliability across various tools is crucial. This includes developing more cost-effective and user-friendly VR solutions to facilitate broader adoption and ensure consistency in test administration. Moreover, combining insights from both traditional and VR-based assessments could provide a more comprehensive evaluation of spatial navigation abilities, ultimately enhancing diagnostic precision and aiding in the differentiation of neurodegenerative disorders. By incorporating VR tools into routine clinical assessments, clinicians can offer more dynamic evaluations that better reflect real-world navigation skills, potentially leading to earlier and more accurate diagnosis of conditions like AD and MCI. Finally, future research should prioritize expanding sample diversity and exploring real-world applications of these tools. By addressing the current gaps and enhancing the practical applicability of these tests, clinicians can better assess and manage cognitive decline in the elderly, leading to more tailored interventions and improved outcomes for patients. This integration of advanced and traditional testing methods will ensure a more robust approach to diagnosing and monitoring cognitive impairments.

## Figures and Tables

**Table 1:** Summary and comparison of spatial navigation tests.

Test	Purpose	Strengths	Profile of Subjects	Utility for Screening Diseases
**Benton’s Judgment of Line Orientation (BJLO)**	Measures spatial orientation and perception	High measurement accuracy; detailed assessment	Generally, for individuals with higher education; useful for patients with MCI	Useful for screening early signs of AD
**Spatial Span subtest (Wechsler Memory Scale)**	Measures spatial short-term memory and manipulation	Widely used; assesses both forward and backward spatial span	Suitable for elderly individuals with varying levels of cognitive decline	Useful in distinguishing between normal aging and early stages of AD
**Corsi Block-Tapping Task (CBT)**	Evaluates spatial memory through replication of block sequences	Reliable measure; adaptable to digital formats	Typically used for individuals with mild to moderate cognitive decline; adaptable to various educational levels	Screening for cognitive deficits related to AD and other forms of dementia
**Money Road-Map Test (MRMT)**	Measures right-left orientation and spatial navigation	Simple administration; useful for assessing right-left confusion	Effective for individuals with neurological conditions affecting spatial orientation	Screening for right-left orientation deficits in conditions like stroke or MCI
**Spatial Orientation in Virtual Environment Test (SOIVET)**	Uses immersive VR to assess spatial orientation and memory	Highly immersive; can simulate real-world scenarios	Suitable for elderly individuals with moderate cognitive decline; requires familiarity with VR	Screening for spatial memory deficits, particularly in MCI and early AD
**Almeria Spatial Memory Recognition Test (ASMRT)**	Assesses spatial memory through virtual room tasks	Easy to administer; suitable for brain imaging studies	Suitable for elderly individuals with mild to moderate cognitive impairment	Useful for screening early AD and distinguishing between MCI and normal aging
**Virtual Supermarket Test (VST)**	Assesses ability to recognize scenes and landmarks	Useful for identifying deficits in scene recognition	Elderly individuals with early cognitive decline; requires basic familiarity with digital environments	Screening for scene recognition deficits in early AD
**Sea Hero Quest (SHQ)**	Assesses spatial navigation in real-world and virtual settings	Good ecological validity; useful for large-scale studies	Suitable for elderly individuals with varying levels of cognitive decline; adaptable to different educational levels	Screening for early cognitive decline and differentiating between normal aging and AD
**Detour Navigation Test (DNT)**	Evaluates spatial navigation with obstacles and detours	Adaptable to various environments; practical for real-world application	Suitable for individuals with moderate cognitive decline; requires basic navigation abilities	Screening for navigation deficits in early AD and other dementias
**Morris Water Maze (MWM)**	Tests spatial learning and memory in virtual and real-world settings	Consistent reliability; effective in assessing hippocampus-dependent spatial navigation	Suitable for elderly individuals with moderate to severe cognitive impairment	Screening for hippocampus-dependent spatial navigation deficits, particularly in AD
**4 Mountains Test (4MT)**	Assesses allocentric spatial memory	Effective in distinguishing MCI and early AD; short duration	Suitable for elderly individuals with early cognitive decline; independent of language skills	Screening for allocentric spatial memory deficits in early AD
**Apple Game**	Measures path integration performance	Designed for older adults; evaluates PI under different spatial cue conditions	Suitable for elderly individuals with early to moderate cognitive decline	Screening for path integration deficits in early AD and other cognitive impairments

**Table 2:** Spatial navigation tests, limitations, and future research suggestions.

Test	Research Gaps	Proposed Future Research Strategies
**Benton’s Judgment of Line Orientation (BJLO)**	Shortened versions have significant error rates; limited validation across diverse populations	Validate shortened versions with larger and more diverse samples; explore potential adaptations for quicker administration
**Spatial Span subtest (Wechsler Memory Scale)**	Challenging for severely impaired participants; lacks validation in large elderly samples	Validate the test with larger elderly samples across different cognitive states; develop simplified instructions for those with severe impairments
**Corsi Block-Tapping Task (CBT)**	Lack of standardization across digital formats; variability in device usage	Standardize digital versions of the test; ensure consistency in device usage and reporting methods; expand research on diverse elderly populations
**Money Road-Map Test (MRMT)**	Limited to right-left discrimination; may not capture broader spatial orientation aspects	Explore the potential for VR adaptations that encompass a broader range of spatial orientation tasks; expand research into the real-world applicability of the test
**Spatial Orientation in Virtual Environment Test (SOIVET)**	High setup cost; limited accessibility	Develop more affordable and accessible versions of the test; validate with diverse elderly populations in different settings
**Almeria Spatial Memory Recognition Test (ASMRT)**	Limited research on sex differences; small sample sizes in some studies	Conduct studies with larger, diverse samples; investigate potential sex differences in spatial memory performance; integrate precise digital measurement tools
**Virtual Supermarket Test (VST)**	Limited applicability outside specific research contexts	Develop more practical versions for wider clinical use; validate the test with larger elderly populations to ensure broader applicability
**Sea Hero Quest (SHQ)**	Inconsistent test-retest reliability; limited research on elderly populations	Improve the test-retest reliability through refined methodologies and performance metrics; expand research involving elderly populations
**Detour Navigation Test (DNT)**	Limited research on elderly; moderate test-retest reliability	Standardize test protocols and performance metrics; expand research focusing on elderly populations to improve reliability and applicability
**Morris Water Maze (MWM)**	Requires specialized equipment; can be physically demanding for elderly	Explore the development of simpler, more accessible versions of the test that are suitable for elderly individuals; expand research on real-world applications
**4 Mountains Test (4MT)**	Requires precise instruction and practice; needs standardization	Simplify administration procedures to make the test more accessible; increase standardization across different testing environments
**Apple Game**	Complex for older participants without training; limited use in research	Validate the test with larger, more diverse elderly samples; refine difficulty levels to ensure broader applicability across different cognitive states
